# Study of Microscale Meniscus Confined Electrodeposition Based on COMSOL

**DOI:** 10.3390/mi12121591

**Published:** 2021-12-20

**Authors:** Fuyue Zhang, Dongjie Li, Weibin Rong, Liu Yang, Yu Zhang

**Affiliations:** 1 School of Measurement and Communication, Harbin University of Science and Technology, Harbin 150080, China; 1920510060@stu.hrbust.edu.cn; 2 Heilongjiang Key Laboratory of Complex Intelligent System and Integration, Harbin University of Science and Technology, Harbin 150069, China; yangliuheu@hrbust.edu.cn (L.Y.); yuzhang@hrbust.edu.cn (Y.Z.); 3 State Key Laboratory of Robotics and System, Harbin Institute of Technology, Harbin 150069, China; rwb@hit.edu.cn

**Keywords:** microscale meniscus, electrodeposition, COMSOL, genetic algorithm

## Abstract

The rate and quality of microscale meniscus confined electrodeposition represent the key to micromanipulation based on electrochemistry and are extremely susceptible to the ambient relative humidity, electrolyte concentration, and applied voltage. To solve this problem, based on a neural network and genetic algorithm approach, this paper optimizes the process parameters of the microscale meniscus confined electrodeposition to achieve high-efficiency and -quality deposition. First, with the COMSOL Multiphysics, the influence factors of electrodeposition were analyzed and the range of high efficiency and quality electrodeposition parameters were discovered. Second, based on the back propagation (BP) neural network, the relationships between influence factors and the rate of microscale meniscus confined electrodeposition were established. Then, in order to achieve effective electrodeposition, the determined electrodeposition rate of 5 × 10^−8^ m/s was set as the target value, and the genetic algorithm was used to optimize each parameter. Finally, based on the optimization parameters obtained, we proceeded with simulations and experiments. The results indicate that the deposition rate maximum error is only 2.0% in experiments. The feasibility and accuracy of the method proposed in this paper were verified.

## 1. Introduction

Electrodeposition is a technique whereby metal ions of the cathode solution are discharged under the action of an electric field and reduced to metal atoms deposited on the cathode substrate [1]. The principle of electrodeposition is applied in the field of micro/nano manufacturing, which can achieve the precision machining of micro and even nano level parts [2,3]. The production and performance analysis of composite coatings can be obtained by the electrodeposition technique [4,5,6]. Electrodeposition based on flat substrates has been applied to additive manufacturing [7,8,9,10]. Meniscus confined electrodeposition (MCED) is a new three-dimensional (3D) printing method that can manufacture a pure metal micro/nanostructure [11]. Hu et al. developed an electrodeposition method that exploits the thermodynamic stability of a microscale or nanoscale liquid meniscus to “write” pure copper and platinum 3D structures of designed shapes and sizes in an ambient air environment [12]. To improve the efficiency of electrodeposition, Lin et al. demonstrated a self-regulated electrodeposition mechanism, which is exploited to realize the parallel process 3D printing by simultaneously printing multiple identical 3D metal microstructures using a nozzle array [13]. Seol et al. presented a 3D printing method that can control the ambient relative humidity and voltage to control the electrodeposition of metal microstructures and fabricate complex metal microstructures [14]. Bhuiyan et al. demonstrated interconnect fabrication by electroless plating on 3D printed electroplated patterns and implemented a hybrid process for printing pure and high conductivity nanocrystalline copper and nickel on flexible polymeric substrates [15,16]. 

The technology of picking up micro-components plays a decisive role in the assembly of complex systems in micro/nanoscale [17]. The traditional method of picking up micro-components with a mechanical manipulation tool can easily cause irreversible damage to the object [18,19]. We proposed a reliable and non-destructive manipulation method for metal micro-components based on electrochemistry [20,21]. However, while picking up by electrochemistry, deposition first occurs in the meniscus area between the manipulating tool and the object. Therefore, the quality and efficiency of the microscale meniscus confined electrodeposition determines the success of subsequent manipulations. Seol et al. pointed out that the ambient relative humidity and voltage have an influence on the deposited metal structure, and the shape and size of the growth structure can be easily controlled by adjusting the growth conditions [14]. To monitor contact points and manufacturing quality, Chen et al. studied the influence of process parameters, including the ion current, microneedle aperture, bias voltage, and solution concentration, on the deposition results [22]. Morsali et al. developed a Multiphysics finite element model to simulate the influence of the ambient relative humidity, nozzle speed, and diameter on the meniscus confined electrodeposition. They pointed out the influence of evaporation on the meniscus deposition and the pipette movement speed under various nozzle diameters [23,24]. However, the microscale meniscus electrodeposition is affected by the electrolyte concentration, the voltage, and the ambient relative humidity. The rate and quality of deposition cannot both be optimal as both factors are complex and non-linear. Thus, the relationships between each influence factor and the electrodeposition rate are difficult to determine with mathematical expressions. 

Hence, in this paper, we use COMSOL Multiphysics to analyze the influence on the microscale meniscus confined electrodeposition. Based on a back propagation (BP) neural network and genetic algorithm approach, the parameters of the microscale meniscus confined electrodeposition are optimized to achieve high-efficiency and -quality electrodeposition.

## 2. Principles of Microscale Confined Meniscus Electrodeposition

Microscale meniscus confined electrodeposition relies on a micropipette containing electrolytes with a microscopic nozzle (20 μm in diameter) as manipulation tool. 

**Figure 1:**
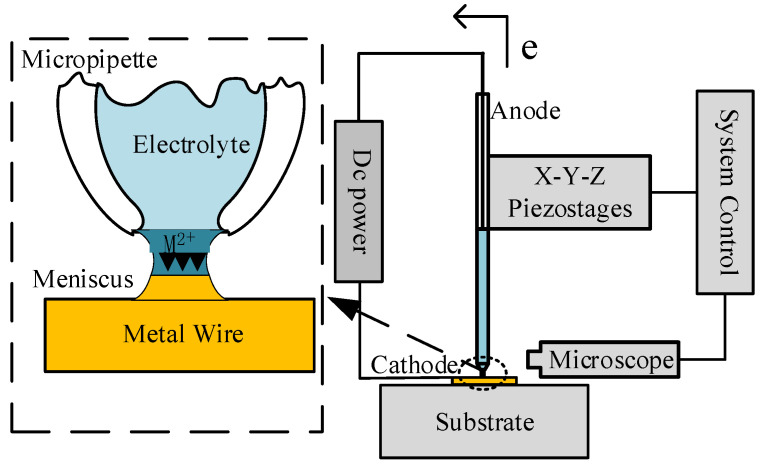
Principles of microscale meniscus confined electrodeposition.

As shown in Figure 1, the pipette is connected to the micro-movement platform and the movement of the pipette is controlled by a computer. The copper microwire is placed over the surface of the conductive substrate. When the copper microwire is approached by the micropipette nozzle, we apply appropriate pressure on the back of the micropipette, to establish a meniscus between the micropipette nozzle and the surface of the micro copper wire. The anode is the copper microwire that is inserted into the electrolyte from the back opening of the micropipette, and the cathode is the surface of the micro copper wire on the conductive substrate. With an electrometer to apply an appropriate voltage, the deposition of copper starts to occur in the meniscus. The electrodeposition process is shown in real time on the computer with the microscope.

## 3. COMSOL Multiphysics Simulation

Many researchers have carried out numerous experiments on electrodeposition [25]. However, because of expensive equipment and materials, if experiments fail, it will result in economic losses. Thus, prior to experiments, finite element simulations are carried out to the extent possible [26]. In this paper, COMSOL Multiphysics is used to simulate the electrodeposition process and analyze the factors influencing the electrodeposition. These include ambient relative humidity, electrolyte concentration, and applied voltage.

### 3.1. Influence of Ambient Relative Humidity on Electrodeposition

Evaporation from the meniscus affects the electrolyte concentration at the tip of the pipette and generates convection, which affects the flow of ions to the cathode [27]. Thus, the ambient relative humidity determines the growth rate and geometrical uniformity of the deposited microstructure. Therefore, it is necessary to find the suitable ambient relative humidity for electrodeposition. With finite element analysis and modeling, we establish a 2D dispersion model of meniscus water evaporation in COMSOL Multiphysics. The borderline condition of Dirichlet is chosen for the pipette nozzle and the nearby main area. The ambient relative humidity at the opening of the pipette is set at 100%, and the ambient relative humidity in the surrounding main area is set at 30% to 80%. The process of water molecule transfer in the meniscus is studied. 

**Figure 2:**
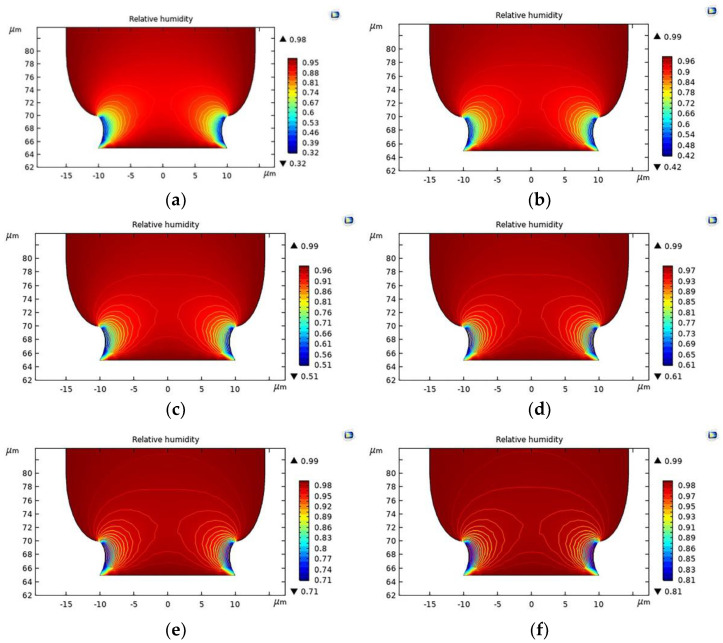
Ambient relative humidity simulation. (**a**) 30% relative humidity; (**b**) 40% relative humidity; (**c**) 50% relative humidity; (**d**) 60% relative humidity; (**e**) 70% relative humidity; (**f**) 80% relative humidity.

The results of the ambient relative humidity simulation are shown in Figure 2, the relative humidity of a, b, c, d, e, and f are 30%, 40%, 50%, 60%, 70%, and 80%, respectively. In the microscale environment, where the relative humidity is too low, the evaporation of electrolytes can easily lead to the formation of crystallites at the pipette nozzle, which will block the pipette and prevent further deposition. If the humidity is too high, water will condense on the tip of the micropipette and the surface of the substrate, thereby diluting the electrolyte in the meniscus and affecting the electrodeposition process. Therefore, the ambient relative humidity should be set at 50–60% to meet the requirements of high-efficiency and -quality deposition.

### 3.2. Electrodeposition Simulation

**Figure 3:**
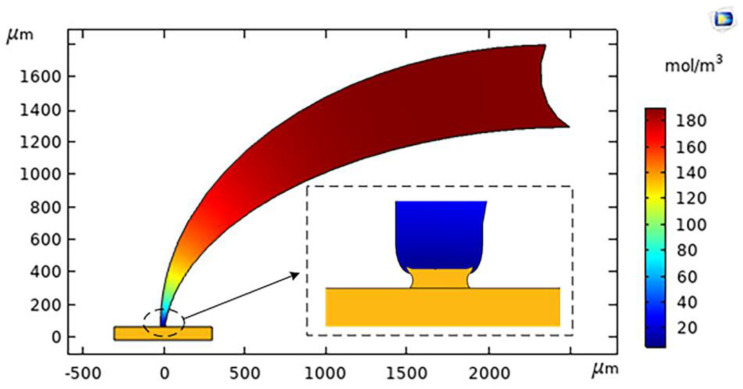
The microscale meniscus confined electrochemical deposition simulation.

With the COMSOL Multiphysics electrodeposition module, the influences of voltage and electrolyte concentration on the microscale meniscus electrodeposition are simulated. As shown in Figure 3, the microscale meniscus confined electrodeposition simulation model is a 2D cross-sectional axisymmetric structure. 

The area in the pipette represents the electrolyte (CuSO_4_ aqueous solution). The yellow rectangle below represents the copper micro wire (the manipulating object), and the lower boundary is the cathode surface. The top end of the pipette is the anode, and the sides of the pipette are isolated. The meniscus between the pipette and the copper microwire is the surface of the contact electrode, which is the electrodeposition portion of the meniscus. Boundary constraints like isolation and symmetry are added at each boundary. The flux for each of the ions in the electrolyte is given by the Nernst–Planck Equation (1),

(1) $$N_{i}=-D_{i}\nabla c_{i}-z_{i}u_{i}Fc_{i}\nabla \phi_{l},$$

where $N_{i}$ denotes the transport vector (mol/(m^2^·s)), $c_{i}$ the concentration in the electrolyte (mol/m^3^), $z_{i}$ the charge for the ionic species, $u_{i}$ the mobility of the charged species (m^2^/(s·j·mol)), *F* Faraday’s constant (A·s/mol), and $\phi_{l}$ the potential in the electrolyte (V).

The anode and cathode boundary conditions are provided by Equations (2) and (3) for electrodeposited copper.

(2) $$N_{{Cu}^{2+}}\cdot n=-\frac{i_{0}}{2F}\left(e^{\frac{1.5F\left(\phi_{s,an}-\phi_{l}-\Delta \phi_{eq}\right)}{RT}}-\frac{c_{{Cu}^{2+}}}{c_{{Cu}^{2+},ref}}e^{-\frac{0.5F\left(\phi_{s,an}-\phi_{l}-\Delta \phi_{eq}\right)}{RT}}\right),$$

(3) $$N_{{Cu}^{2+}}\cdot n=-\frac{i_{0}}{2F}\left(e^{\frac{1.5F\left(\phi_{s,cat}-\phi_{l}-\Delta \phi_{eq}\right)}{RT}}-\frac{c_{{Cu}^{2+}}}{c_{{Cu}^{2+},ref}}e^{-\frac{0.5F\left(\phi_{s,cat}-\phi_{l}-\Delta \phi_{eq}\right)}{RT}}\right),$$

where n denotes the normal vector to the boundary.

During the electrodeposition on the cathode and the dissolution on the anode, we assume that current efficiency is 100%. This means that the model does not consider possible side reactions, so the total reaction is:

(4) $${Cu}^{2+}+2e^{-}=Cu$$

During the electrodeposition process, the cathode boundary keeps moving, indicating the evolution of the electrodeposition process.

#### 3.2.1. Influence of Voltage on Electrodeposition Rate and Quality

Since the electrical voltage directly affects the overpotential, it has a great influence on the deposition rate and quality [18]. In this paper, we keep the electrolyte concentration of 0.500 mol/L and the voltage variation range from 0.05 V to 0.35 V.

**Figure 4:**
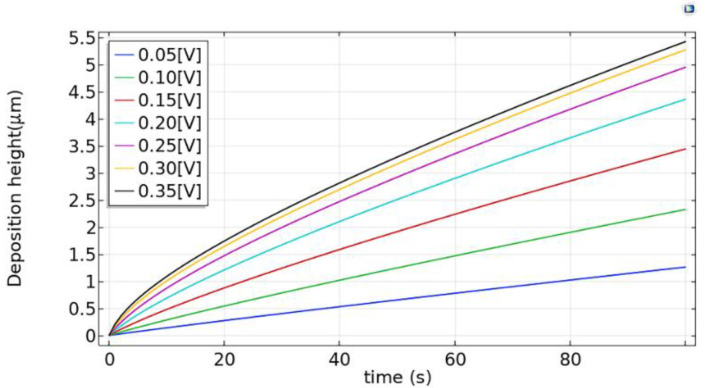
Influence of voltage on electrodeposition rate.

By calculation and post-processing, the simulation results regarding the influence of the voltage on the electrodeposition rate are shown in Figure 4.

As the voltage increases, the electromigration rate and charge conversion rate increase, so that the electrodeposition rate increases as the voltage increases. From 0.05 to 0.25 V, the deposition rate naturally increases with the voltage, but when the voltage exceeds 0.25 V, the electrodeposition rate increases slowly. In addition, excessive voltage can easily cause uneven copper deposition and hydrogen reduction reaction, which will cause the deposition rate at the edge to be higher than the center and generate bubbles. Therefore, the high voltage is detrimental to the quality of the deposited copper. As a result, the appropriate voltage is 0.15–0.25 V in the experiments, which can provide a higher electrodeposition rate and quality.

#### 3.2.2. Influence of Electrolyte Concentration on Electrodeposition Rate

**Figure 5:**
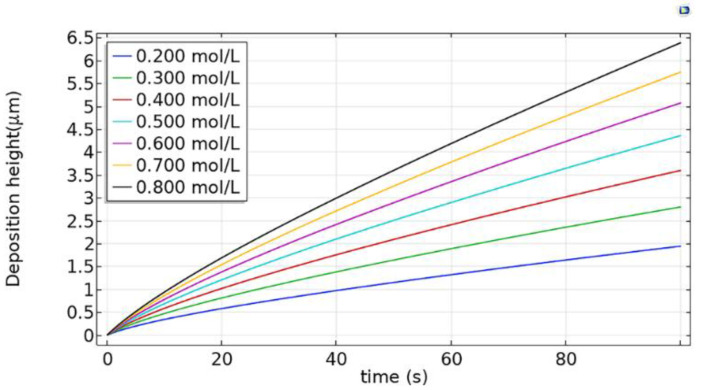
Influence of electrolyte concentration on electrodeposition rate.

In the ion-deficient state, the electrolyte concentration plays a key role in the electrodeposition rate. If more copper is precipitated, in the pipette nozzle, the copper ions of the electrolyte will not be reconstituted in time and an ion-deficient area of copper will be produced [18]. Therefore, it is beneficial to study the influence of electrolyte concentration on the electrodeposition rate for the deposition mechanism. In this paper, the influence of electrolyte concentration on the deposition rate is simulated. The results of simulation are shown in Figure 5. The anode loading voltage is 0.20 V, and the electrolyte concentration is 0.200–0.800 mol/L.

Figure 5 shows that the rate of electrodeposition increases with the increase of electrolyte concentration. Therefore, high concentration is the primary choice for efficient deposition. However, when the electrolyte concentration is too high, due to the evaporation of water, crystal precipitation and clogging are likely to occur the pipette nozzle. The low concentration electrolytes can alleviate the clogging problem. However, when the electrolyte concentration is too low, the electrodeposition rate is also too low, and the deposition time will increase. Moreover, a low electrolyte concentration can more easily cause the appearance of a metal ion deficient area. Therefore, in order to satisfy the requirements of high efficiency and non-clogging of the pipette during manipulation, the electrolyte concentration should be set to 0.400–0.600 mol/L.

## 4. Microscale Meniscus Confined Electrodeposition Parameter Optimization

The relationship between the various factors that affect the rate of electrodeposition is complex and non-linear [28], so it is difficult to determine with mathematical expressions. To solve this problem, based on the BP neural network, we establish the mapping relationships of the various factors and the electrochemical deposition rate. The determined electrodeposition rate is the target value, and the genetic algorithm is used to optimize each parameter, so as to find the best applicable voltage and electrolyte concentration.

After analyzing the influence factors, because the ambient relative humidity is not the main factor that influences the deposition rate, it is set to 50%. The voltage and electrolyte concentration are chosen as two major factors influencing the input values of the neural network model. The critical values for each parameter are defined according to the simulation results shown in Table 1.

### 4.1. Constructing Mapping Relationships

The BP neural network structure is 2-5-1. The data from the selected sample must be universal and not aggregated to a certain value. Hence, 200 sets of simulation data are used as the input and output sample data for the neural network model. In order to facilitate the processing of the experimental results, 20 sets of data are taken out in an orderly and uniform manner as the test sample data. The remaining data are used as the network model training sample data. After the network training is completed, 200 sets of data are filtered, the sample values are compared in the results, and the resulting figures drawn. 

**Figure 6:**
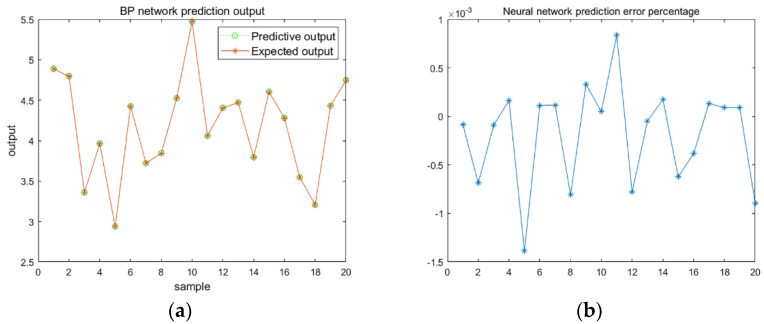
Neural network prediction results. (**a**) BP network prediction output; (**b**) Neural network prediction error percentage.

Figure 6 shows the comparison curve of the relationship of the neural network detection sample data with the output value of the network model. The sample detection data are uniformly selected. Figure 6 shows that the predictive output value of the neural network after training is relatively consistent with the expected output and the maximum error is only 0.1487%. The neural network has good accuracy and can predict the electrodeposition rate.

### 4.2. Parameter Optimization Based on BP Neural Network and Genetic Algorithm

The BP neural network has strong nonlinear mapping ability and self-adaptive ability, but the training speed of the BP neural network is slow. Moreover, the global search ability of the BP neural network is weak, so it is easy to fall into local minima [29]. The genetic algorithm has a strong global search capability, and the evolution process of each generation of the population can easily obtain the global optimal solution [30]. The neural network and genetic algorithm are combined to establish a microscale system for optimizing meniscus electrodeposition parameters, which can globally optimize process parameters to obtain the best combination [31]. 

The optimization of the neural network parameters and the genetic algorithm is mainly divided into the formation and adjustment of the neural network BP and the optimization of the parameters of the genetic algorithm.

**Figure 7:**
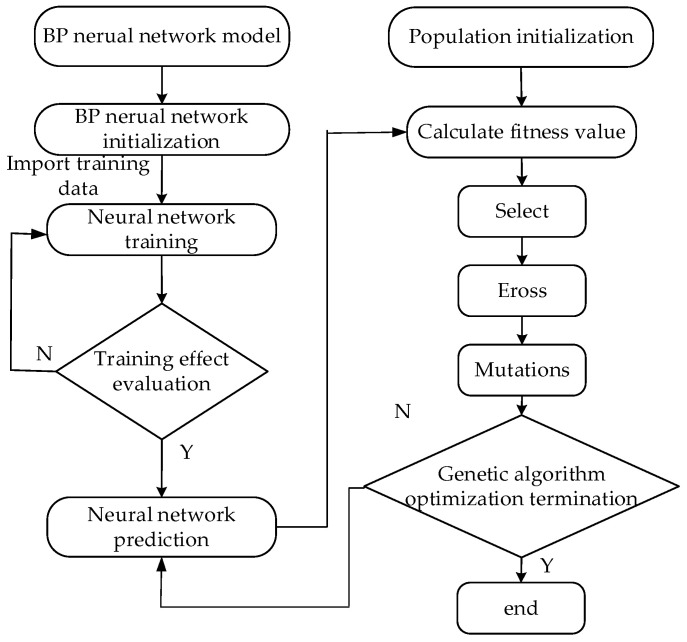
BP neural network and genetic algorithm.

The workflow is shown in Figure 7. As an optimization process, each individual value in the variable group is provided by the genetic algorithm as the input of the BP neural network, and the BP neural network model is trained to the output value. The value of the target is the determined electrodeposition rate. The value of the target is subtracted from the output value of the neural network, and the absolute value is calculated to obtain the fitness value of the individual. Then, the algorithm performs selection, crossover, and mutation operations to obtain the next generation. After iteration and evolutionary computation, until the convergence condition is satisfied, the optimal solution is obtained.

To achieve efficient deposition and improve the efficiency of micromanipulation, the electrodeposition rate 5.0 × 10^−8^ m/s is set as the target value of the genetic algorithm, and the upper and lower boundaries of the voltage and electrolyte concentration are set. The size of the genetic algorithm group is set to 40. The crossover probability is set to 0.4, and the mutation probability is set to 0.2. The generation of iterative optimization is set to 100.

After 100 generations, the convergence conditions are satisfied, and satisfactory optimization results are obtained. The voltage is 0.2186 V, and the electrolyte concentration is 0.5480186 mol/L.

## 5. Simulations and Experiments

Due to the limited accuracy of voltage and concentration in the simulations and experiments, the optimized electrodeposition voltage is 0.22 V and the electrolyte concentration is 0.550 mol/L. To verify the optimized accuracy, the optimized process parameters are simulated and calculated.

**Figure 8:**
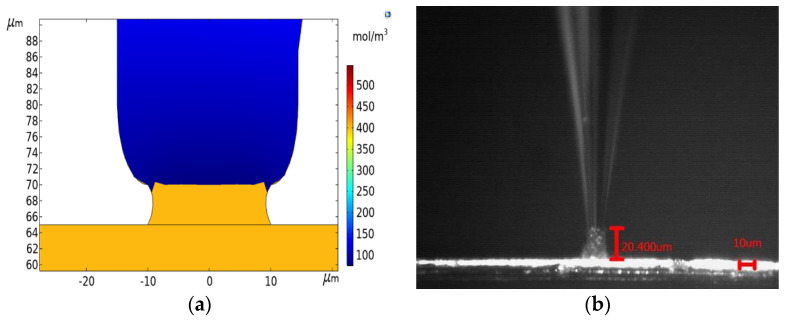
Simulation and experiments. (**a**) Electrodeposition simulation result; (**b**) Electrodeposition experiment result.

The simulation result is shown in Figure 8a. After 100 s, the height difference between the center and the edge is small. The deposition height is 5.0398 μm according to the simulation result with COMSOL. Hence, the microscale electrodeposition rate is 5.0398 × 10^−8^ m/s and the error is only 0.8%, which realizes the precise control of the electrodeposition rate and verifies the accuracy of the genetic algorithm optimization.

In order to prove the effectiveness of the optimization, the microscale meniscus confined electrodeposition experiments were carried out. The ambient relative humidity is 50%, the deposition voltage is 0.22 V, and the electrolyte concentration is 0.550 mol/L. The micro pipette containing the electrolyte is attached to the anode, and the conductive substrate on which the micro copper wire is placed is attached to the cathode. The pipette is moved above the micro copper wire. The electrolyte at the nozzle of the pipette has a concave shape due to the surface tension, so that the electrolyte at the nozzle of the pipette forms a convex surface by applying external pressure above the pipette. The pipette is moved down to make the electrolyte have contact with the object, to establish a meniscus between the micropipette nozzle and the surface of the micro copper wire. When the electrometer detects a microcurrent in the circuit, the deposited copper starts to occur in the meniscus. The electrodeposition process is shown real time on the computer with the microscope. In order to improve the measurement accuracy, we have increased the time of the experiment.

The deposition height is measured with Image Analysis 9. Five sets of experiments and measurements are carried out. After 400 s, the experimental result is shown in Figure 8b, and the results of the deposition height are shown in Table 2. In the third set of experiment, the deposition height is 20.400 μm, the deposition rate is 5.100 × 10^−8^ m/s, and the maximum error is only 2.0%. The simulation and assumptions are verified.

## 6. Conclusions

In this paper, we analyzed the factors influencing the microscale meniscus confined electrodeposition with COMSOL Multiphysics and found the appropriate range of variables used during the experiments. The ambient relative humidity is 40–60%, the deposition voltage is 0.15–0.25 V, and the electrolyte concentration is 0.400–0.600 mol/L. The ambient relative humidity is 50% and the target value of electrodeposition rate is set to 5. 0 × 10^−8^ m/s. Based on the BP neural network and genetic algorithm, the microscale meniscus confined electrodeposition parameters are optimized, and the voltage and electrolyte concentration are 0.22 V and 0.550 mol/L, respectively. Finally, the optimized parameters are used for simulations and experiments. The maximum error of the experiment is only 2.0%, and the deposition quality is ideal. The accuracy and effectiveness of the proposed method are verified. In this paper, high-efficiency and -quality microscale meniscus confined electrodeposition is obtained. This method promotes the application of microscale meniscus confined electrodeposition within the field of three-dimensional (3D) printing, picking up micro-components, and composite coatings.

## Figures and Tables

**Table 1:** Value range of influencing factors.

Factors	Minimum	Maximum
voltage (V)	0.15	0.25
Concentration (mol/L)	0.400	0.600

**Table 2:** The results of electrodeposition experiment.

Experiment	1	2	3	4	5
Deposition height (μm)	19.807	20.340	20.400	19.776	20.236
Deposition rate (m/s)	4.952 × 10^−8^	5.085 × 10^−8^	5.100 × 10^−8^	4.944 × 10^−8^	5.059 × 10^−8^
Error	0.96%	1.7%	2.0%	1.12%	1.18%

## References

[1] Low C., Wills R., Walsh F.C. (2006). Electrodeposition of composite coatings containing nanoparticles in a metal deposit. Surf. Coat. Technol..

[2] Kim S.P., Choe H.C. (2019). Functional elements coatings on the plasma electrolytic oxidation-treated Ti-6Al-4V alloy by rlectrochemical precipitation method. J. Nanosci. Nanotechnol..

[3] Huang T., Liu Z.Q., Huang G.S., Liu R., Mei Y.F. (2014). Grating-structured metallic microsprings. Nanoscale.

[4] Tseluikin V.N., Koreshkova A.A. (2014). Deposition of zinc–carbon nanotube composite coatings in the pulse-reverse mode. Russ. J. Appl. Chem..

[5] Gobinda G., Bhupendra J., Khagendra T., Soo W.L. (2017). Effect of ultrasonic nanocrystal surface modification on properties of electrodeposited Ni and Ni-SiC composite coatings. J. Mater. Eng. Perform..

[6] Lanzutti A., Lekka M., Leitenburg C., Fedrizzi L. (2019). Effect of pulse current on wear behaviour of Ni matrix micro- and nano-SiC composite coatings at room and elevated temperature. Tribol. Int..

[7] Farahani R.D., Chizari K., Therriault D. (2014). Three-dimensional printing of freeform helical microstructures: A review. Nanoscale.

[8] Momotenko D., Page A., Adobes M., Patrick R.U. (2016). Write-read 3D patterning with a dual-channel nanopipette. ACS Nano.

[9] Yi Z.R., Lei Y., Zhang X.Y., Chen Y.N., Guo J.J., Xu G.J., Yu M.F., Cui P. (2017). Ultralow flexural properties of copper microhelices fabricated via electrodeposition-based three-dimensional direct-writing technology. Nanoscale.

[10] Lei Y., Zhang X.Y., Xu D.D., Yu M.F., Guo J.J. (2018). Dynamic “scanning-mode” meniscus confined electrodepositing and micropatterning of individually addressable ultraconductive copper line arrays. J. Phys. Chem. Lett..

[11] Wang C., Bhuiyan E.H., Moreno S., Minary-Jolandan M. (2020). Direct-Write Printing Copper-Nickel (Cu/Ni) Alloy with Controlled Composition from a Single Electrolyte Using Co-Electrodeposition. ACS Appl. Mater. Interfaces.

[12] Hu J., Yu M.F. (2010). Meniscus-confined three-dimensional electrodeposition for direct writing of wire bonds. Science.

[13] Lin Y.P., Zhang Y., Yu M.F. (2019). Parallel process 3D metal microprinting. Adv. Mater. Technol..

[14] Seol S.K., Kim D., Lee S., Kim J.H., Chang W.S. (2015). Electrodeposition-based 3D printing of metallic microarchitectures with controlled internal structures. Small.

[15] Bhuiyan M., Moreno S., Wang C., Minary-Jolandan M. (2021). Interconnect Fabrication by Electroless Plating on 3D-Printed Electroplated Patterns. ACS Appl. Mater. Interfaces.

[16] Bhuiyan M., Behroozfar A., Daryadel S., Moreno S., Minary-Jolandan M. (2019). A Hybrid Process for Printing Pure and High Conductivity Nanocrystalline Copper and Nickel on Flexible Polymeric Substrates. Sci. Rep..

[17] Lofrotn M., Avci E. (2019). Development of a novel modular compliant gripper for manipulation of micro-objects. Micromachines.

[18] Almeida A., Andrews G., Jaiswal D., Hoshino K. (2019). The actuation mechanism of 3D printed flexure-based robotic microtweezers. Micromachines.

[19] Hao G.B., Zhu J.X. (2019). Design of a monolithic double-slider based compliant gripper with large displacement and anti-buckling ability. Micromachines.

[20] Li D.J., Xu J.Y., Rong W.B., Yang L. (2020). Simulation of picking up metal microcomponents based on electrochemistry. Micromachines.

[21] Li D.J., Wang M.R., Xu J.Y., Yang L., Zhang Y. (2021). Simulation of metal microcomponents picking up by electrochemical based on ABAQUS. Modern Phys. Lett. B.

[22] Chen Y.L., Wang Y., Wang Y., Ju B.F. (2021). Meniscus-confined electrodeposition of metallic microstructures with in-process monitoring of surface qualities. J. Precis. Eng..

[23] Morsali S., Soheil D., Zhong Z. (2017). Multi-physics simulation of metal printing at micro/nanoscale using meniscus-confined electrodeposition: Effect of nozzle speed and diameter. J. Appl. Phys..

[24] Morsali S., Soheil D., Zhong Z. (2017). Multi-physics simulation of metal printing at micro/nanoscale using meniscus-confined electrodeposition: Effect of environmental humidity. J. Appl. Phys..

[25] Volgin V.M., Lyubimov V.V., Davydov A.D. (2016). Modeling and numerical simulation of electrochemical micromachining. Chem. Eng. Sci..

[26] Brant A.M., Sundaram M.M., Kamaraj A.B. (2015). Finite Element Simulation of Localized Electrochemical Deposition for Maskless Electrochemical Additive Manufacturing. J. Manuf. Sci. Eng..

[27] Suryavanshi A.P., Yu M.F. (2007). Electrochemical fountain pen nanofabrication of vertically grown platinum nanowires. Nanotechnology.

[28] Kul M., Oskay K.O., Erden F., Akca E., Katirci R., Koksal E., Akinci E. (2020). Effect of process parameters on the electrodeposition of zinc on 1010 steel: Central composite design optimization. Int. J. Electrochem. Sci..

[29] Zhang L., Wang F., Sun T., Xu B. (2018). A constrained optimization method based on BP neural network. Neural Comput. Appl..

[30] Ding S.F., Su C.Y., Yu J.Z. (2011). An optimizing BP neural network algorithm based on genetic algorithm. Artif. Intell. Rev..

[31] Han J.X., Ma M.Y., Wang K. (2021). Product modeling design based on genetic algorithm and BP neural network. Neural Comput. Appl..

